# A Social Media–Based Mindfulness Psycho-Behavioral Intervention (MCARE) for Patients With Acute Coronary Syndrome: Randomized Controlled Trial

**DOI:** 10.2196/48557

**Published:** 2024-02-20

**Authors:** Huijing Zou, Sek Ying Chair, Bilong Feng, Qian Liu, Yu Jia Liu, Yu Xin Cheng, Dan Luo, Xiao Qin Wang, Wei Chen, Leiqing Huang, Yunyan Xianyu, Bing Xiang Yang

**Affiliations:** 1 School of Nursing Wuhan University Wuhan China; 2 The Nethersole School of Nursing The Chinese University of Hong Kong Hong Kong China (Hong Kong); 3 Zhongnan Hospital of Wuhan University Wuhan China; 4 Renmin Hospital of Wuhan University Wuhan China

**Keywords:** acute coronary syndrome, psychological distress, depression, anxiety, mindfulness, mindfulness-based intervention, quality of life, risk factors, cardiac rehabilitation, social media

## Abstract

**Background:**

Psychological distress is common among patients with acute coronary syndrome (ACS) and has considerable adverse impacts on disease progression and health outcomes. Mindfulness-based intervention is a promising complementary approach to address patients’ psychological needs and promote holistic well-being.

**Objective:**

This study aims to examine the effects of a social media–based mindfulness psycho-behavioral intervention (MCARE) on psychological distress, psychological stress, health-related quality of life (HRQoL), and cardiovascular risk factors among patients with ACS.

**Methods:**

This study was a 2-arm, parallel-group randomized controlled trial. We recruited 178 patients (mean age 58.7, SD 8.9 years; 122/178, 68.5% male) with ACS at 2 tertiary hospitals in Jinan, China. Participants were randomly assigned to the MCARE group (n=89) or control group (n=89). The 6-week intervention consisted of 1 face-to-face session (phase I) and 5 weekly WeChat (Tencent Holdings Ltd)–delivered sessions (phase II) on mindfulness training and health education and lifestyle modification. The primary outcomes were depression and anxiety. Secondary outcomes included psychological stress, HRQoL, and cardiovascular risk factors (ie, smoking status, physical activity, dietary behavior, BMI, blood pressure, blood lipids, and blood glucose). Outcomes were measured at baseline (T0), immediately after the intervention (T1), and 12 weeks after the commencement of the intervention (T2).

**Results:**

The MCARE group showed significantly greater reductions in depression (T1: β=–2.016, 95% CI –2.584 to –1.449, Cohen *d*=–1.28, *P*<.001; T2: β=–2.089, 95% CI –2.777 to –1.402, Cohen *d*=–1.12, *P*<.001) and anxiety (T1: β=–1.024, 95% CI –1.551 to –0.497, Cohen *d*=–0.83, *P*<.001; T2: β=–0.932, 95% CI –1.519 to –0.346, Cohen *d*=–0.70, *P*=.002). Significantly greater improvements were also observed in psychological stress (β=–1.186, 95% CI –1.678 to –0.694, Cohen *d*=–1.41, *P*<.001), physical HRQoL (β=0.088, 95% CI 0.008-0.167, Cohen *d*=0.72, *P*=.03), emotional HRQoL (β=0.294, 95% CI 0.169-0.419, Cohen *d*=0.81, *P*<.001), and general HRQoL (β=0.147, 95% CI 0.070-0.224, Cohen *d*=1.07) at T1, as well as dietary behavior (β=0.069, 95% CI 0.003-0.136, Cohen *d*=0.75, *P*=.04), physical activity level (β=177.542, 95% CI –39.073 to 316.011, Cohen *d*=0.51, *P*=.01), and systolic blood pressure (β=–3.326, 95% CI –5.928 to –0.725, Cohen *d*=–1.32, *P*=.01) at T2. The overall completion rate of the intervention (completing ≥5 sessions) was 76% (68/89). Positive responses to the questions of the acceptability questionnaire ranged from 93% (76/82) to 100% (82/82).

**Conclusions:**

The MCARE program generated favorable effects on psychological distress, psychological stress, HRQoL, and several aspects of cardiovascular risk factors in patients with ACS. This study provides clues for guiding clinical practice in the recognition and management of psychological distress and integrating the intervention into routine rehabilitation practice.

**Trial Registration:**

Chinese Clinical Trial Registry ChiCTR2000033526; https://www.chictr.org.cn/showprojEN.html?proj=54693

## Introduction

Acute coronary syndrome (ACS), an acute manifestation of ischemic heart disease, has become a major public health problem worldwide [1]. In China, ischemic heart disease affects approximately 11.4 million people [2], and there remains a rising trend in the morbidity and mortality of ACS [3], thus posing a huge challenge for the health care system.

Psychological distress, such as depression and anxiety, is highly prevalent in patients with ACS [4] and has considerable adverse impacts on disease progression and health outcomes. Clear evidence supports that psychological distress is associated with functional disability, reduced health-related quality of life (HRQoL), and increased risks of cardiac events [5,6]. Nonetheless, current health care practice has paid inadequate attention to the recognition and management of psychological distress. A growing consensus advocates that psychological distress is a crucial risk factor of ACS that should be addressed in disease management [5,7,8]. The American Heart Association has recommended mindfulness-based intervention as a promising complementary approach to promoting psychological health and well-being for patients with cardiovascular disease [8]. Emerging evidence has proven its benefits in improving a wide range of psychological and physical outcomes [9,10], including among patients with ischemic heart disease [11], indicating its potential as an additional supplement to conventional cardiac care.

Another practice gap in China is that optimal rehabilitation and effective control of cardiovascular risk factors are rarely achieved due to insufficient awareness and competency of health care professionals, inadequate workforce, and limited resources [12]. The health care system primarily focuses on in-hospital treatments of acute attacks of ACS and neglects the posthospital management of risk factors. An investigation of 991 hospitals in China showed that only 228 (23%) hospitals provided center-based cardiac rehabilitation services, which were mainly distributed in urban areas (89.1%) [2].

In recent years, mobile health or eHealth technologies are increasingly being used to improve the availability, feasibility, and affordability of posthospital care with inspiring results in promoting medication adherence, lifestyle changes, and health outcomes [13,14]. With the popularization and widespread coverage of mobile internet access, WeChat (Tencent Holdings Ltd), a free smartphone app, has become one of the most popular and widely used social media in China. It provides various services, including instant messaging, voice and video calls, microblogging and subscription services, and web-based banking. WeChat may provide an unprecedented approach to addressing the shortage of posthospital care, considering its wide population reach and powerful peripheral functions.

This study proposed a WeChat-based mindfulness psycho-behavioral intervention (MCARE), which integrated mindfulness training with health education and lifestyle modification to assist patients in managing risk factors. This randomized controlled trial (RCT) aimed to examine the effects of the MCARE program on psychological distress (primary outcomes), psychological stress, HRQoL, and cardiovascular risk factors (secondary outcomes) among patients with ACS.

## Methods

### Study Design and Participants

This study was a 2-arm, 1:1 parallel-group RCT. Participants were recruited using convenience sampling from June to September 2020 at the wards of the cardiology department of 2 public tertiary hospitals in Jinan, China. Inclusion criteria were as follows: (1) age 18 to 75 years; (2) clinical diagnosis of ACS (including unstable angina and acute myocardial infarction); (3) ability to read, understand, communicate, and complete questionnaires in Chinese; and (4) possession of an operational smartphone and an active WeChat account. Participants were excluded if they (1) were in the active state of myocardial infarction or receiving open-heart surgical treatment; (2) had a clinical diagnosis of serious physical comorbidities, for example, cancer and renal failure; (3) had psychiatric disorders; (4) had cognitive impairments as documented in the health records; or (5) were currently participating in other interventions.

The sample size was calculated following a power analysis approach using G*Power 3.1 (Universität Düsseldorf). Considering a significance level of 0.05; a statistical power of 80%; an effect size of Cohen *d*=0.50 for primary outcomes, namely depression and anxiety [15]; and a potential attrition rate of 20%, in all,160 participants (80 per group) were required.

### Ethical Considerations

Ethics approvals were obtained from the Joint Chinese University of Hong Kong-New Territories East Cluster Clinical Research Ethics Committee (2019.323) and the Ethics Committee of the School of Nursing, Shandong University (2019-R-017). Participants gave informed consent to participate in the study before taking part. All information and data from participants were anonymous and confidentially was guaranteed by coding participants with unique identification numbers (eg, 001). All participants received a small red envelope sent via WeChat upon completion of each intervention session and after each data collection to acknowledge the time and effort they dedicated to participating in the study.

### Procedure

All procedures of the RCT were completed in the same manner in 2 research hospitals. Two research assistants (registered nurses) were hired and received training on participant recruitment; data collection; and the rationale, principles, and procedure of the study. The research assistants approached eligible participants, introduced the study details, and invited them to participate in this study. Then they obtained written informed consent and performed a baseline assessment.

Participants were randomly allocated into either the intervention or control groups at a 1:1 ratio using a sequence of block randomized numbers generated by an independent statistician using a computer procedure with a block size of 4. The group allocation was concealed by placing the random sequence in a sequentially numbered, opaque, and sealed envelope by an independent person. The research assistants were blinded to group allocation.

### Interventions

**Figure 1:**
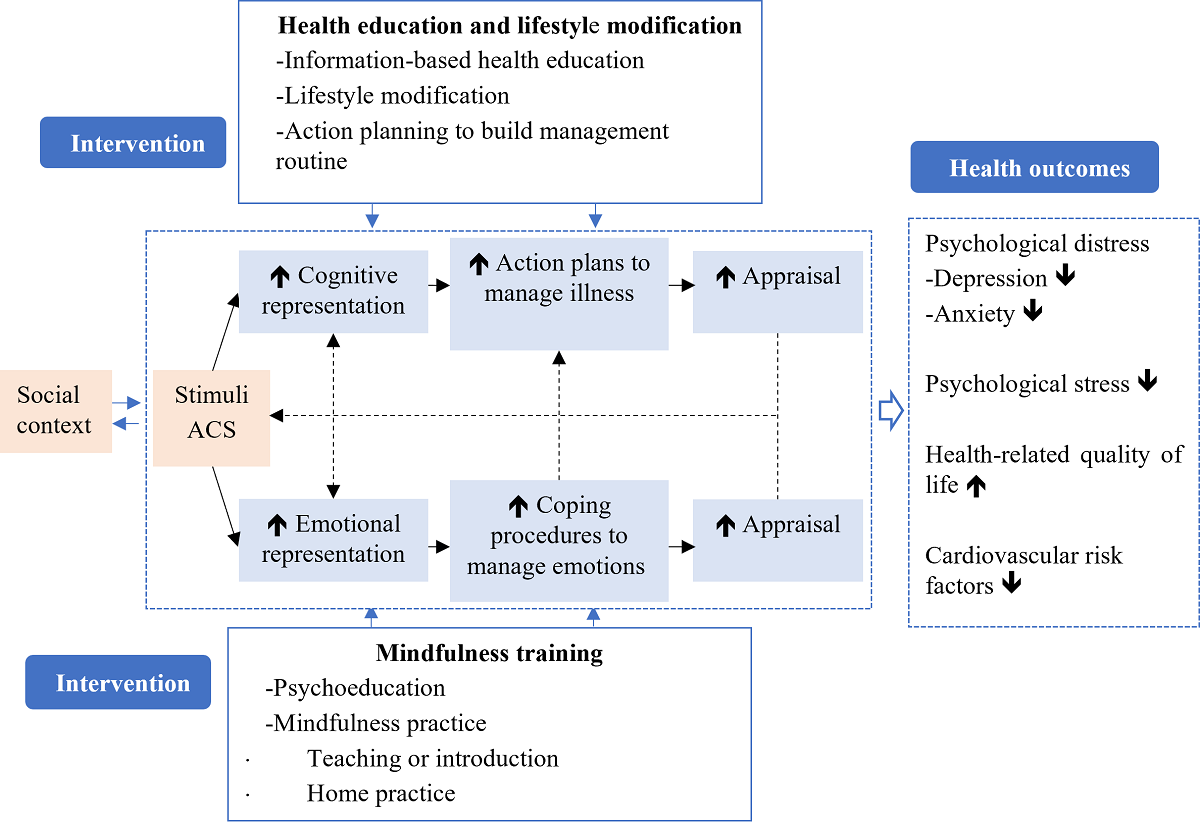
The conceptual framework underpinning the mindfulness psycho-behavioral intervention program. ACS: acute coronary syndrome.

The MCARE program was developed on the basis of the Common-Sense Model of Self-Regulation theory [16], clinical guideline recommendations [7,17], and experimental studies [8,10,11]. It aimed to promote psychological and physical well-being by targeting emotional and cognitive procedures for managing health threats. Figure 1 illustrates the conceptual framework underpinning the MCARE program.

**Figure 2:**
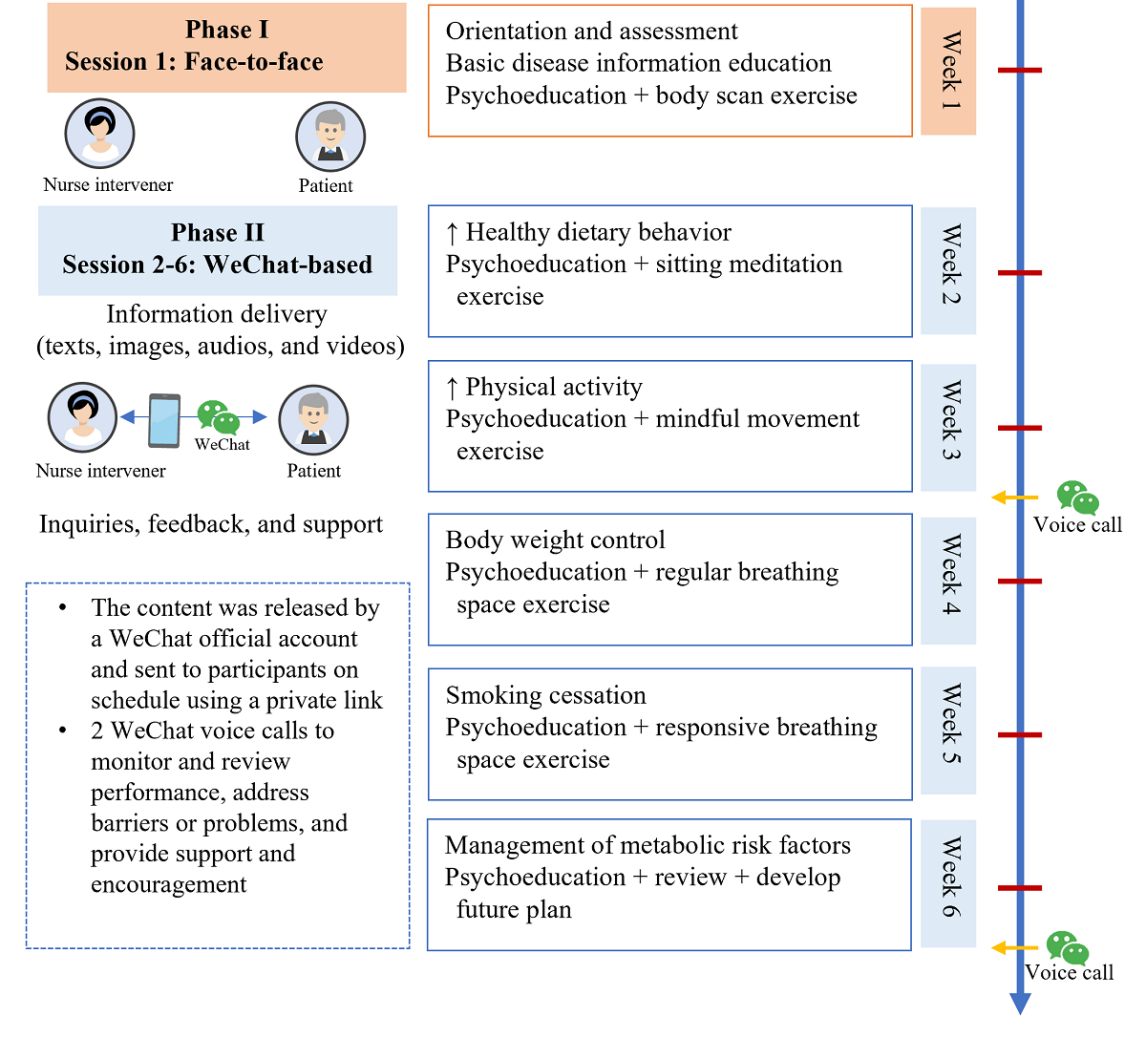
The delivery plan of the mindfulness psycho-behavioral intervention program.

The MCARE program comprised 6 weekly sessions and each session focused on a thematic topic in mindfulness training and disease management (Table S1 in Multimedia Appendix 1). Mindfulness training topics included simple awareness, mindfulness of breath, mindfulness of the body, dealing with thoughts, dealing with difficulties, and maintenance. Disease management topics were basic disease information, healthy dietary behavior, physical activity, body weight control, smoking cessation, and management of metabolic risk factors. Additionally, participants were required to perform home-based mindfulness practice for 10 to 20 minutes per day for 6 days per week. Figure 2 summarizes the detailed delivery plan. The first session was delivered face-to-face during hospitalization (phase I) and the following 5 sessions were delivered using WeChat after discharge (phase II). Following the third and sixth sessions, participants received a voice call to monitor and review their performance, address barriers or problems, and provide support and encouragement. Participants also received an information handbook and audio and video guides on mindfulness practice.

Participants in the control group received routine medical treatment and care before hospital discharge. To control the nonspecific effect of attention, the control group also received WeChat contacts at the same frequency as the intervention group. The contents of the contacts were limited to general information about the importance of managing stress and risk factors without providing specific advice and strategies.

### Measures

The research assistants collected data at baseline (T0), immediately after the intervention (T1), and 12 weeks following the commencement of the intervention (T2). A self-designed data collection sheet was used to assess baseline sociodemographic and clinical characteristics through patient interviews and medical record reviews.

Primary outcomes were psychological distress, including depression and anxiety, which were assessed using the 9-item Patient Health Questionnaire (PHQ-9) [18] and the 7-item Generalized Anxiety Disorder (GAD-7) [19], respectively. Secondary outcomes were psychological stress, HRQoL, and cardiovascular risk factors. Psychological stress was assessed using the 10-item Perceived Stress Scale [20]. HRQoL was measured using the HeartQoL questionnaire [21], which comprises 14 items capturing disease-specific HRQoL in physical (10 items) and emotional (4 items) dimensions. Cardiovascular risk factors included (1) smoking status, as measured by self-reported 7-day smoking history [22]; (2) physical activity, as measured using the International Physical Activity Questionnaire-Short Form [23]; (3) dietary behavior, as assessed using the nutrition subscale of the Health-Promoting Lifestyle Profile-II [24]; (4) BMI, as calculated using the formula: *BMI = body weight (in kilograms) / height (in meters) squared*; (5) blood pressure (BP); (6) blood lipid profiles; and (7) blood glucose. Body weight, height, and BP were obtained by anthropometric measures, and blood lipids and blood glucose were measured via laboratory tests of fasting blood samples.

The acceptability of the MCARE program among participants in the MCARE group was also measured at T1 using a self-developed dichotomous questionnaire (positive ratings ≥80% are considered acceptable). Additionally, the completion of the intervention, performance of home mindfulness practice, difficulties or problems encountered by participants, and adverse events were collected.

Baseline (T0) assessment was conducted at the wards, and follow-up assessments (T1 and T2) were completed via telephone interviews. After the first face-to-face session, the assessors would schedule the telephone interview for each participant. Participants were reminded to return to the research hospitals or go to a nearby accredited hospital to complete fasting blood tests at T2. If the participants did not answer the telephone call, they would contact the participant 3 times at different periods of a day within 1 week. If none of these telephone calls reached the participant, the participant would be considered lost to follow-up.

### Statistical Analysis

Statistical analyses were performed using IBM SPSS (version 25.0). All statistical tests were 2-tailed tests and statistical significance was set at 0.05. Appropriate descriptive statistics were calculated to summarize the participant characteristics and outcomes. The intention-to-treat principle was applied in outcome analysis. The generalized estimating equation (GEE) analyses were performed to examine the differential changes of each outcome variable across 3 data collection time points between intervention and control groups. Baseline characteristics and outcomes between intervention and control groups were compared and no significant differences were observed, therefore only the crude GEE models were performed without adjustment of confounding variables. A dummy variable (group) was set to represent the MCARE group with the control group as the reference. To represent time differences, the baseline (T0) was set as the reference, and another 2 dummy variables, T1 and T2, were assigned to correspond to immediate postintervention and 12 weeks. The interaction terms of the group-by-time dummy variables, group×T1 and group×T2, were included in the GEE models to assess the overall differences in the outcomes between the 2 groups at T1 and T2. Effect sizes were estimated using Cohen *d* statistic for continuous outcomes and odds ratio for binary outcomes.

We calculated the percentage of missing data for each outcome (9.0% to 9.9%) and compared baseline sociodemographic and clinical characteristics between participants who had completed all observations and those who had at least 1 missing observation. Additionally, to examine the effects of the missing data on the estimation of intervention effects we conducted a sensitivity analysis using completed case analysis. In the completed case analysis, only participants who had completed all assessments at T0, T1, and T2 were included in the estimation of intervention effects. We also examined the correlation between participants’ completion of intervention and dosage of home mindfulness practice on changes in outcome variables between T1 and T0 by performing Pearson correlation analysis.

## Results

### Participant Recruitment and Retention

**Figure 3:**
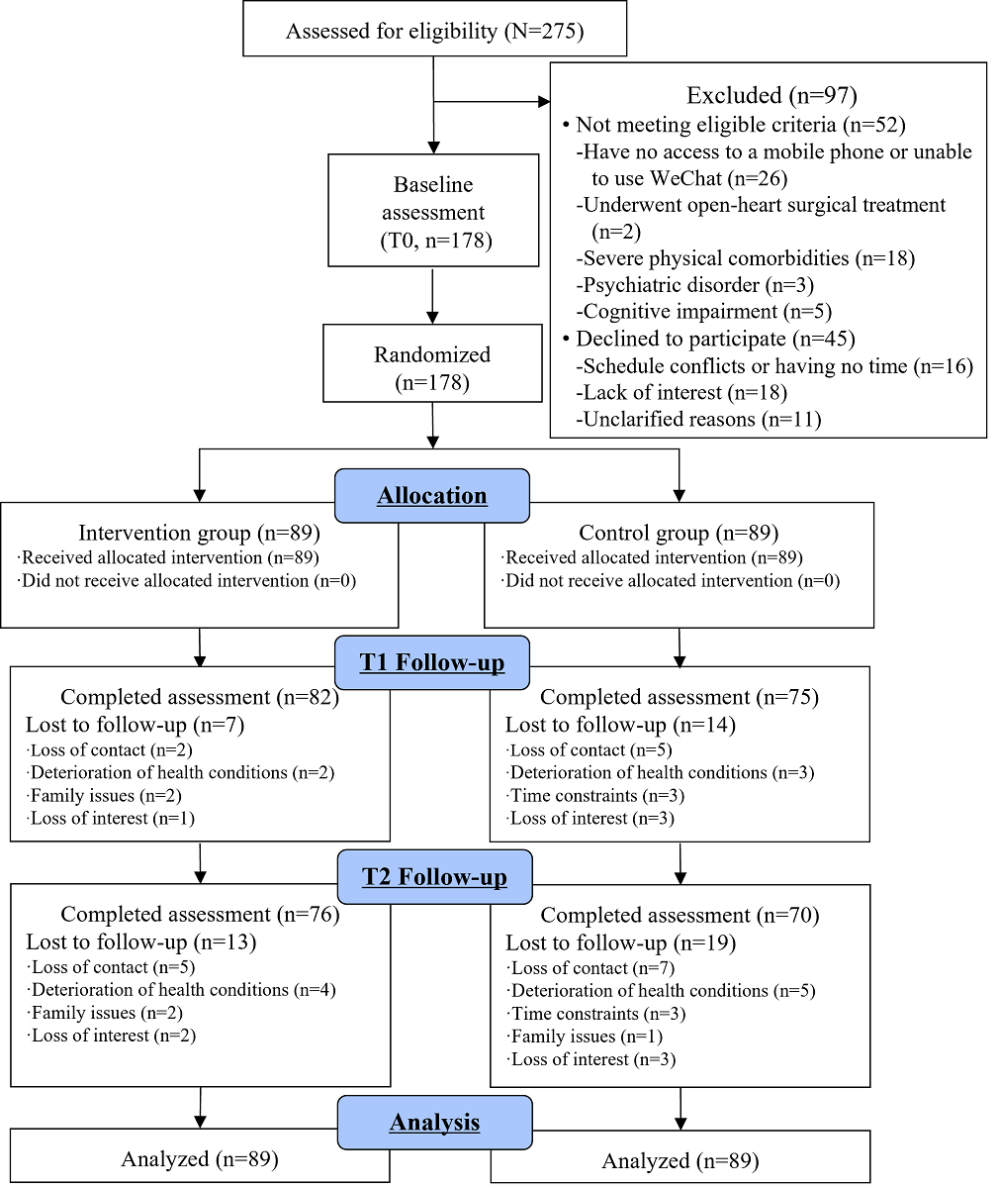
Flow chart of participant recruitment, allocation, intervention delivery, follow-up, and data analysis of the study.

Of the 275 patients with ACS screened for eligibility, 52 patients did not meet the eligibility criteria and 45 patients declined to participate. Finally, 178 participants were enrolled (Figure 3). At follow-up, 157 (88.2%) participants completed T1 assessment, and 146 (82.0%) participants completed T2 assessment. Cardiovascular risk factors were measured only at T0 and T2 with a total of 356 observations, and 32 (9.0%) were missing. All the other outcome variables had a total of 534 observations across 3 study time points with 53 (9.9%) missing observations. There was no significant difference in baseline characteristics between participants who completed all observations (n=146) and who had at least 1 missing observation (n=32; Table S2 in Multimedia Appendix 1). No adverse events related to the intervention were reported.

### Baseline Characteristics

Table 1 presents the detailed baseline characteristics of participants and the intervention and control groups were well-matched. Mean age of the participants was 58.7 (SD 8.9) years, ranging from 28 to 75 years. The majority of them were male (122/178, 68.5%), married (172/178, 96.6%), received a secondary education or less (147/178, 82.6%), and had a New York Heart Association class of I or II (148/178, 83.1%). Over half of the participants were experiencing ACS for the first time (104/178, 58.4%) and did not receive percutaneous transluminal coronary intervention (99/178, 55.6%). The mean scores for PHQ-9 and GAD-7 were 5.66 (SD 3.30) and 5.38 (SD 2.97), respectively. Over half of the participants had depressive symptoms (PHQ-9 score ≥5; 107/178, 60.1%) and anxiety symptoms (GAD-7 score ≥5; 95/178, 53.4%), indicating psychological distress is highly prevalent.

**Table 1:** Baseline characteristics and outcomes of all participants and by the mindfulness psycho-behavioral intervention.

Characteristics		All (n=178)	Intervention group (n=89)	Control group (n=89)	*P* value	
Age (years), mean (SD)		58.73 (8.90)	59.48 (8.95)	58.02 (8.90)	.28	
**Sex, n (%)**						.52
	Male	122 (68.5)	59 (66.3)	63 (70.8)		
	Female	56 (31.5)	30 (33.7)	26 (29.2)		
**Marital status, n (%)**						.41
	Married	172 (96.6)	87 (97.8)	85 (95.5)		
	Single, separated, divorced, or widowed	6 (3.4)	2 (2.2)	4 (4.5)		
**Educational level, n (%)**						.49
	Primary education	34 (19.1)	20 (22.5)	14 (15.7)		
	Secondary education	113 (63.5)	55 (61.8)	58 (65.2)		
	Tertiary education	31 (17.4)	14 (15.7)	17 (19.1)		
**Employment status, n (%)**						.35
	Employed	66 (37.1)	30 (33.7)	36 (40.4)		
	Unemployed, farmer, retired, or freelance	112 (62.9)	59 (66.3)	53 (59.6)		
**Monthly income (CNY ¥^a^), n (%)**						.75
	<1500	27 (15.2)	12 (13.5)	15 (16.8)		
	1500-3000	68 (38.2)	36 (40.4)	32 (36)		
	≥3000	83 (46.6)	41 (46.1)	42 (47.2)		
**Episode of ACS^b^, n (%)**						.13
	First	104 (58.4)	57 (64)	47 (52.8)		
	Recurrent	74 (41.6)	32 (36)	42 (47.2)		
**Percutaneous transluminal coronary intervention, n (%)**						.05
	Yes	79 (44.4)	33 (37.1)	46 (51.7)		
	No	99 (55.6)	56 (62.9)	43 (48.3)		
**NYHA^c^ functional classification, n (%)**						.37
	I	83 (46.6)	45 (50.6)	38 (42.7)		
	II	65 (36.5)	30 (33.7)	35 (39.3)		
	III	21 (11.8)	8 (9.0)	13 (14.6)		
	IV	9 (5.1)	6 (6.7)	3 (3.4)		
LVEF^d^ (%), mean (SD)		56.61 (10.69)	57.00 (8.66)	56.21 (10.57)	.59	
Number of comorbidities, mean (SD)		1.13 (0.87)	1.06 (0.82)	1.20 (0.92)	.26	
Depression (PHQ-9^e^), mean (SD)		5.66 (3.30)	5.37 (3.26)	5.96 (3.34)	.24	
Anxiety (GAD-7^f^), mean (SD)		38 (2.97)	5.25 (2.96)	5.48 (2.93)	.59	
Psychological stress (PSS-10^g^), mean (SD)		16.24 (4.54)	16.35 (4.66)	16.12 (4.45)	.74	
**HRQoL^h^ (HeartQoL), mean (SD)**						
	Physical HRQoL	1.89 (0.61)	1.91 (0.63)	1.87 (0.58)	.67	
	Emotional HRQoL	2.18 (0.70)	2.19 (0.74)	2.17 (0.65)	.92	
	General HRQoL	1.98 (0.56)	1.99 (0.58)	1.96 (0.54)	.70	
**Smoking status, n (%)**						.86
	Nonsmoker	133 (74.7)	66 (74.2)	67 (75.3)		
	Current smoker	45 (25.3)	23 (25.8)	22 (24.7)		
Physical activity (IPAQ-SF^i^) (MET·min/wk), mean (SD)		712.78 (665.45)	727.85 (686.95)	697.70 (646.78)	.76	
Dietary behavior (HPLP-II^j^ nutrition subscale), mean (SD)		2.43 (0.37)	2.46 (0.38)	2.40 (0.35)	.26	
BMI (kg/m^2^), mean (SD)		25.10 (3.72)	24.98 (3.66)	25.10 (3.20)	.81	
Systolic BP^k^ (mm Hg), mean (SD)		126.06 (17.29)	126.64 (18.08)	125.48 (16.55)	.66	
Diastolic BP (mm Hg), mean (SD)		75.92 (10.24)	75.47 (10.03)	76.36 (10.48)	.56	
LDL-C^l^ (mmol/L), mean (SD)		2.24 (0.82)	2.22 (0.82)	2.26 (0.82)	.76	
HDL-C^m^ (mmol/L), median (IQR)		1.09 (0.91, 1.30)	1.00 (0.90, 1.25)	1.12 (0.92, 1.37)	.20	
TG^n^ (mmol/L), median (IQR)		1.36 (1.00-1.99)	1.36 (0.98-2.00)	1.36 (1.02-2.04)	.75	
TC^o^ (mmol/L), mean (SD)		4.00 (1.07)	3.90 (1.02)	4.10 (1.12)	.22	
FBG^p^ (mmol/L), median (IQR)		5.47 (4.74-6.51)	5.56 (4.89-6.93)	5.22 (4.69-6.28)	.31	

^a^CNY ¥: Chinese yuan, US $1=CNY ¥6.90 at the time of the study (2020).

^b^ACS: acute coronary syndrome.

^c^NYHA: New York Heart Association.

^d^LVEF: left ventricular ejection fraction.

^e^PHQ-9: 9-item Patient Health Questionnaire.

^f^GAD-7: 7-item Generalized Anxiety Disorder.

^g^PSS-10: 10-item Perceived Stress Scale.

^h^HRQoL: health-related quality of life.

^i^IPAQ-SF: International Physical Activity Questionnaire-Short Form.

^j^HPLP-II: Health-Promoting Lifestyle Profile-II.

^k^BP: blood pressure.

^l^LDL-C: low-density lipoprotein cholesterol.

^m^HDL-C: high-density lipoprotein cholesterol.

^n^TG: triglyceride.

^o^TC: total cholesterol.

^p^FBG: fasting blood glucose.

### Intervention Effects

There were significant time-by-group interaction effects on psychological distress with moderate to large effects at both T1 and T2 (Table 2). Participants in the intervention group demonstrated significantly greater reductions in depression (T1: β=–2.016, 95% CI –2.584 to –1.449, Cohen *d*=–1.28, *P*<.001; T2: β=–2.089, 95% CI –2.777 to –1.402, Cohen *d*=–1.12, *P*<.001) and anxiety (T1: β=–1.024, 95% CI –1.551 to –0.497, Cohen *d*=–0.83, *P*<.001; T2: β=–0.932, 95% CI –1.519 to –0.346, Cohen *d*=–0.70, *P*=.002) than those in the control group.

**Table 2:** Generalized estimating equation analyses for the comparison of depression and anxiety of the mindfulness psycho-behavioral intervention program^a^.

Outcome and time point			Intervention group		Control group		Group effect^b^					Time effect^c^					Group×time effect^d^				
							β (95% CI)		*P* value		β (95% CI)			*P* value		β (95% CI)			Effect size		*P* value
**Depression (PHQ-9^e^, total score range 0-27)^f^**																					
	T0	5.37 (3.26)		5.96 (3.34)		–0.584 (–1.548 to 0.379)		.24		N/A^g^			N/A		N/A			N/A		N/A	
	T1	3.72 (3.03)		6.32 (3.19)		N/A		N/A		0.365 (–0.060 to 0.790)			.09		–2.016 (–2.584 to –1.449)			–1.28		<.001	
	T2	2.53 (2.39)		5.20 (3.02)		N/A		N/A		–0.755 (–1.255 to –0.256)			.003		–2.089 (–2.777 to –1.402)			–1.12		<.001	
**Anxiety (GAD-7^h^, total score range 0-21)^f^**																					
	T0	5.25 (2.96)		5.48 (2.93)		–0.236 (–1.096 to 0.625)		.59		N/A			N/A		N/A			N/A		N/A	
	T1	4.07 (2.38)		5.33 (2.71)		N/A		N/A		–0.150 (–0.555 to 0.255)			.47		–1.024 (–1.551 to –0.497)			–0.83		<.001	
	T2	1.63 (2.03)		2.80 (2.45)		N/A		N/A		–2.683 (–3.121 to –2.245)			<.001		–0.932 (–1.519 to –0.346)			–0.70		.002	

^a^The control group (group =0) and the baseline measurement (time =0) were set as the reference categories in the generalized estimating equation model and its corresponding null variables.

^b^Group effect was defined as group differences at baseline between intervention and control groups.

^c^Time effect at T1 is defined as change of scores for the control group at T1 compared with T0; T2 is defined as change of scores for the control group at T2 compared with T0.

^d^Group×time effect at T1 defined as additional change of scores for the intervention group compared with the control group at T1; T2 defined as additional change of scores for the intervention group compared with the control group at T2. Effect sizes were estimated using Cohen *d* statistic for continuous outcomes and odds ratio for binary outcomes.

^e^PHQ-9: 9-item Patient Health Questionnaire.

^f^Intervention and control group data are presented as mean (SD).

^g^N/A: not applicable.

^h^GAD-7: 7-item General Anxiety Disorder.

Compared with control group, the intervention group demonstrated significantly greater improvements in psychological stress (β=–1.186, 95% CI –1.678 to –0.694, Cohen *d*=–1.41, *P*<.001), physical HRQoL (β=0.088, 95% CI 0.008-0.167, Cohen *d*=0.72, *P*=.03), emotional HRQoL (β=0.294, 95% CI 0.169-0.419, Cohen *d*=0.81, *P*<.001), and general HRQoL (β=0.147, 95% CI 0.070-0.224, Cohen *d*=1.07, *P*<.001) at T1 (Table 3). However, the significant effects were only sustained for psychological stress (β=–1.268, 95% CI –1.992 to –0.544, Cohen *d*=–1.17, *P*=.001) and emotional HRQoL (*β*=0.249, 95% CI 0.102-0.395, Cohen *d*=0.62, *P*=.001) but not for physical HRQoL and general HRQoL at T2. For cardiovascular risk factors, the intervention group showed significantly greater improvements in dietary behavior (β=0.069, 95% CI 0.003-0.136, Cohen *d*=0.75, *P*=.04), physical activity level (β=177.542, 95% CI –39.073 to 316.011, Cohen *d*=0.51, *P*=.01), and systolic BP (β=–3.326, 95% CI –5.928 to –0.725, Cohen *d*=–1.32, *P*=.01) at T2 (Table 4). No significant group-by-time interaction effects were observed for other outcomes (all *P*>.05). The sensitivity analysis showed consistent results in the directions of the GEE regression coefficients (Table S3 in Multimedia Appendix 1).

**Table 3:** Generalized estimating equation analyses for the comparison of psychological stress and health-related quality of life of the mindfulness psycho-behavioral intervention program^a^.

Outcome and time point			Intervention group		Control group		Group effect^b^					Time effect^c^					Group×time effect^d^				
							β (95% CI)		*P* value		β (95% CI)			*P* value		β (95% CI)			Effect size		*P* value
**Psychological stress (PSS-10^e^, total score range 0-40)^f^**																					
	T0	16.35 (4.66)		16.12 (4.45)		0.225 (–1.106 to 1.555)		.74		N/A^g^			N/A		N/A			N/A		N/A	
	T1	14.59 (4.71)		15.55 (4.59)		N/A		N/A		–0.577 (–0.950 to –0.204)			.002		–1.186 (–1.678 to –0.694)			–1.41		<.001	
	T2	14.00 (4.52)		15.04 (4.60)		N/A		N/A		–1.081 (–1.538 to –0.624)			<.001		–1.268 (–1.992 to –0.544)			–1.17		.001	
**Physical HRQoL (Physical HeartQoL^h^, subscale score range 0-30)^f^**																					
	T0	1.91 (0.63)		1.87 (0.58)		0.039 (–0.139 to 0.217)		.66		N/A			N/A		N/A			N/A		N/A	
	T1	2.11 (0.62)		1.98 (0.58)		N/A		N/A		0.110 (0.048 to 0.172)			<.001		0.088 (0.008 to 0.167)			0.72		.03	
	T2	2.13 (0.61)		2.09 (0.58)		N/A		N/A		0.213 (0.131 to 0.295)			<.001		0.004 (–0.099 to 0.107)			0.12		.94	
**Emotional HRQoL (Emotional HeartQoL, subscale score range 0-12)^f^**																					
	T0	2.19 (0.74)		2.17 (0.65)		0.011 (–0.193 to 0.215)		.91		N/A			N/A		N/A			N/A		N/A	
	T1	2.52 (0.63)		2.21 (0.61)		N/A		N/A		0.036 (–0.062 to 0.134)			.47		0.294 (0.169 to 0.419)			0.81		<.001	
	T2	2.55 (0.63)		2.29 (0.55)		N/A		N/A		0.115 (–0.001 to 0.232)			.05		0.249 (0.102 to 0.395)			0.62		.001	
**General HRQoL (General HeartQoL, total score range 0-42)^f^**																					
	T0	1.99 (0.58)		1.96 (0.54)		0.031 (–0.133 to 0.195)		.71		N/A			N/A		N/A			N/A		N/A	
	T1	2.23 (0.54)		2.05 (0.51)		N/A		N/A		0.089 (0.029 to 0.148)			.003		0.147 (0.070 to 0.224)			1.07		<.001	
	T2	2.25 (0.53)		2.15 (0.48)		N/A		N/A		0.185 (0.111 to 0.260)			<.001		0.073 (–0.021 to 0.167)			0.47		.13	

^a^The control group (group =0) and the baseline measurement (time =0) were set as the reference categories in the generalized estimating equation model and its corresponding null variables.

^b^Group effect was defined as group differences at baseline between intervention and control groups.

^c^Time effect at T1 is defined as change of scores for the control group at T1 compared with T0; T2 is defined as change of scores for the control group at T2 compared with T0.

^d^Group×time effect at T1 defined as additional change of scores for the intervention group compared with the control group at T1; T2 defined as additional change of scores for the intervention group compared with the control group at T2. Effect sizes were estimated using Cohen *d* statistic for continuous outcomes and odds ratio for binary outcomes.

^e^PSS-10: 10-item Perceived Stress Scale.

^f^Intervention and control group data are presented as mean (SD).

^g^N/A: not applicable.

^h^HRQoL: health-related quality of life.

**Table 4:** Generalized estimating equation analyses for the comparison of cardiovascular risk factors of the mindfulness psycho-behavioral intervention program^a^.

Outcome and time point			Intervention group		Control group		Group effect^b^			Time effect^c^				Group×time effect^d^				
							β (95% CI)	*P* value		β (95% CI)		*P* value		β (95% CI)		Effect size		*P* value
**Smoking status (current smoker)^e^**																		
	T0	23 (25.8)		22 (24.7)		0.059 (–0.617 to 0.736)		.86	N/A^f^		N/A		N/A		N/A		N/A	
	T2	6 (7.9)		9 (12.9)		N/A		N/A	–0.800 (–1.358 to –0.242)		.005		–0.603 (–1.532 to 0.327)		0.55		.20	
**Physical activity (IPAQ-SF^g^, MET•min/week)^h^**																		
	T0	727.85 (686.95)		697.70 (646.78)		30.152 (–164.766 to 225.069)		.76	N/A		N/A		N/A		N/A		N/A	
	T2	881.41 (605.83)		673.71 (541.13)		N/A		N/A	–23.988 (–127.430 to 79.454)		.65		177.542 (39.073 to 316.011)		0.51		.01	
**Dietary behavior (HPLP-II^i^ nutrition subscale, total score range 9-36)^h^**																		
	T0	2.46 (0.38)		2.40 (0.35)		0.061 (–0.045 to 0.168)		.26	N/A		N/A		N/A		N/A		N/A	
	T2	2.88 (0.36)		2.75 (0.34)		N/A		N/A	0.353 (0.309 to 0.397)		<.001		0.069 (0.003 to 0.136)		0.75		.04	
**BMI (kg/m^2^)^h^**																		
	T0	24.98 (3.66)		25.10 (3.20)		–0.121 (–1.126 to 0.883)		.81	N/A		N/A		N/A		N/A		N/A	
	T2	24.38 (3.59)		24.90 (3.11)		N/A		N/A	–0.203 (–0.557 to 0.150)		.26		–0.403 (–0.835 to 0.030)		–0.46		.07	
**Systolic BP^j^ (mmHg)^h^**																		
	T0	126.64 (18.08)		125.48 (16.55)		1.157 (–3.906 to 6.221)		.65	N/A		N/A		N/A		N/A		N/A	
	T2	121.97 (14.44)		124.14 (16.37)		N/A		N/A	–1.340 (–3.073 to 0.393)		.13		–3.326 (–5.928 to –0.725)		–1.32		.01	
**Diastolic BP (mmHg)^h^**																		
	T0	75.47 (10.03)		76.36 (10.48)		–0.888 (–3.884 to 2.108)		.56	N/A		N/A		N/A		N/A		N/A	
	T2	73.12 (9.29)		75.10 (9.24)		N/A		N/A	–1.260 (–2.546 to 0.027)		.06		–1.094 (–2.772 to 0.584)		–0.67		.20	
**LDL-C^k^ (mmol/L)^h^**																		
	T0	2.22 (0.82)		2.26 (0.82)		–0.037 (–0.276 to 0.202)		.76	N/A		N/A		N/A		N/A		N/A	
	T2	2.14 (0.75)		2.16 (0.79)		N/A		N/A	–0.100 (–0.199 to –0.001)		.05		0.025 (–0.112 to 0.162)		0.07		.72	
**HDL-C^l^ (mmol/L)^m^**																		
	T0	1.00 (0.90-1.25)		1.12 (0.92-1.37)		–0.121(–0.247 to 0.006)		.06	N/A		N/A		N/A		N/A		N/A	
	T2	1.18 (0.95-1.37)		1.15 (1.00-1.45)		N/A		N/A	0.043 (–0.025 to 0.112)		.21		0.043 (–0.034 to 0.121)		–0.04		.27	
**TG^n^ (mmol/L)^m^**																		
	T0	1.36 (0.98-2.00)		1.36 (1.02, 2.04)		–0.051(–0.411 to 0.309)		.78	N/A		N/A		N/A		N/A		N/A	
	T2	1.26 (0.87-1.96)		1.25 (0.99-1.90)		N/A		N/A	–0.104 (–0.245 to 0.037)		.15		–0.015 (–0.185 to 0.155)		–0.27		.86	
**TC^o^ (mmol/L)^h^**																		
	T0	3.90 (1.02)		4.10 (1.12)		–0.195 (–0.508 to 0.117)		.22	N/A		N/A		N/A		N/A		N/A	
	T2	3.96 (0.92)		4.07 (0.93)		N/A		N/A	–0.025 (–0.157 to 0.107)		.71		0.080 (–0.096 to 0.257)		0.15		.37	
**FBG^p^ (mmol/L)^m^**																		
	T0	5.56 (4.89-6.93)		5.22 (4.69-6.28)		0.246 (–0.292 to 0.784)		.37	N/A		N/A		N/A		N/A		N/A	
	T2	5.35 (4.80-6.49)		5.21 (4.61-6.27)		N/A		N/A	–0.169 (–0.327 to –0.012)		.04		–0.101 (–0.363 to 0.160)		–0.19		.45	

^a^The control group (group =0) and the baseline measurement (time =0) were set as the reference categories in the generalized estimating equation model and its corresponding null variables.

^b^Group effect was defined as group differences at baseline between intervention and control groups.

^c^Time effect at T1 is defined as change of scores for the control group at T1 compared with T0; T2 is defined as change of scores for the control group at T2 compared with T0.

^d^Group×time effect at T1 defined as additional change of scores for the intervention group compared with the control group at T1; T2 defined as additional change of scores for the intervention group compared with the control group at T2. Effect sizes were estimated using Cohen *d* statistic for continuous outcomes and odds ratio for binary outcomes.

^e^Intervention and control group data are presented as n (%).

^f^N/A: not applicable.

^g^IPAQ-SF: International Physical Activity Questionnaire-Short Form.

^h^Intervention and control group data are presented as mean (SD).

^i^HPLP-II: health-promoting lifestyle profile-II.

^j^BP: blood pressure.

^k^LDL-C: low-density lipoprotein cholesterol.

^l^HDL-C: high-density lipoprotein cholesterol.

^m^Intervention and control group data are presented as median (IQR).

^n^TG: triglyceride.

^o^TC: total cholesterol.

^p^FBG: fasting blood glucose.

### Intervention Adherence

The overall attrition rate of this study was 18% (32/178; intervention group: 13/89, 15% and control group: 19/89, 21%). The completion rate for each intervention session ranged from 68.5% (61/89) to 100% (89/89) and the overall completion rate (defined as completing at least 5 of the 6 intervention sessions) was 76% (68/89). Further analysis showed the number of completed sessions was significantly and positively correlated with changes in depression (*r*=0.324, *P*=.003), psychological stress (*r*=0.224, *P*=.04), emotional HRQoL (*r*=0.224, *P*=.04), and general HRQoL (*r*=0.279, *P*=.01) at T1 (Table S4 in Multimedia Appendix 1), suggesting that adherence to the intervention may influence the intervention effects. The average frequency of home mindfulness practice ranged from 2.2 (SD 2.0) to 3.7 (SD 1.7) times per week and the total average amount was 19.0 (SD 8.9; range 1 to 38) times during the 6 weeks, which was much less than the designed dosage. In addition, the frequency of home mindfulness practice was significantly and positively correlated with changes in depression (*r*=0.865, *P*<.001), anxiety (*r*=0.626, *P*<.001), psychological stress (*r*=0.353, *P*=.001), emotional HRQoL (*r*=0.497*, P*<.001), and general HRQoL (*r*=0.399, *P*<.001) at T1 (Table S4 in Multimedia Appendix 1).

### Acceptability of the Intervention

At T1, a total of 82 (92%) of 89 participants in the intervention group completed the acceptability questionnaire. Positive responses to the questions ranged from 93% (76/82) to 100% (82/82; Table S5 in Multimedia Appendix 1), indicating high satisfaction with the intervention. Furthermore, 13 (15%) participants reported difficulties or problems in applying intervention skills or strategies in daily life over the 6-week intervention, including lack of a suitable environment to apply the skills and strategies (n=6), lack of a quiet environment to concentrate for home mindfulness practice (n=5), lack of time for home mindfulness practice (n=3), and physical discomfort (n=3).

## Discussion

### Principal Findings

This study provided evidence for the effects of a social media–based intervention for patients with ACS. The MCARE program significantly improved psychological distress in terms of depression and anxiety at immediate postintervention and 12-week follow-up. Furthermore, the MCARE program has significant effects on psychological stress, HRQoL, dietary behavior, physical activity, and systolic BP.

The findings were supported by previous reports that mindfulness-based interventions [11,25] and health education [26] had significant effects on reducing depression and anxiety for patients with cardiovascular disease. Mindfulness training together with health education mainly targeted promoting awareness of and response to the feelings, emotions, and bodily sensations caused by the physical and psychological distress and increasing disease management knowledge and skills. Therefore, the MCARE program was assumed to have meaningful effects on psychological distress for patients with ACS. Moreover, the benefits of the MCARE program on psychological distress were likely to be sustained for a short-term period from immediate postintervention to 12-week follow-up. This might be explained by the residual gains of mindfulness skills and disease management knowledge and skills from the MCARE program. The present-focused mindfulness practice may generate a short-term, sustainable, beneficial effect on improving emotional regulation skills to facilitate participants to cope with difficult situations and lead to reduced psychological distress.

The MCARE program also significantly improved psychological stress and HRQoL, which is consistent with previous systematic reviews of mindfulness-based interventions [27] and educational interventions [28] for patients with ischemic heart disease. The MCARE program could help participants cope with their condition and thus reduce mental and emotional distress, which in turn contributed to the improvement of HRQoL, particularly in the emotional dimension.

Previous research rarely reported the effects of mindfulness-based interventions on cardiovascular risk factors. This study demonstrated the promising effects of the MCARE program on dietary behavior, physical activity, and systolic BP for patients with ACS, providing a valuable reference for future research. Health education would help patients understand the etiology, development, duration, and prognosis of their illness and learn how to manage the risk factors [29], and mindfulness training would increase their awareness of managing health threats, which together, in turn, might have empowered them to eat healthier and be more physically active. The enhanced knowledge and awareness about the illness, together with regular BP surveillance and behavioral change might have contributed to improved BP control.

The findings suggested the MCARE program had nonsignificant effects on smoking status, BMI, blood lipid profiles, and blood glucose. This might be due to that the MCARE program only provided general education and support without more effective strategies such as targeted and direct medical cessation therapy for smokers [30] and targeted weight loss strategy for patients who are overweight or obese. Blood lipid and glucose control are important targets of ACS management, which may mostly rely on adherence to core cardioprotective medications that lower blood lipid and glucose. Complementary interventions via the promotion of a healthy lifestyle, providing education on disease knowledge, and regular monitoring may facilitate blood lipid and glucose control; however, it may take a long time to observe a significant improvement in the concentration of blood lipid and glucose.

### Limitations

This study has several limitations. First, this study was carried out in the Chinese population and used a convenience sampling method, which may lead to selection bias, thus limiting the representativeness of the study population and the generalizability of findings. In order to improve the representativeness of the study population, multicenter studies conducted in various regions and diverse settings are warranted. Second, the measure of the majority of outcomes relied on participants’ self-reporting, although the instruments adopted in the study were reliable and validated. The subjective data may be subject to self-reporting and recall bias. Third, this study only evaluated the intervention effects at immediate postintervention and 12-week follow-up, which was unable to explore the sustainability of the intervention effects over the long term. Furthermore, we did not evaluate the consistent use of the intervention and analyze its association with health outcomes including cardiometabolic status. Thus, the patterns of use and the relationships between the use of intervention and health outcomes require further exploration in studies with a larger sample size and a longer follow-up period. Fourth, due to the scope of this study, we did not examine whether the MCARE program supported the conceptual framework or explore whether the key concepts related to the framework would explain the possible mechanism of the intervention effects. Last, this study did not assess all potential confounding factors that may impact the psychological distress and secondary outcomes, such as the baseline cardiovascular risk of participants by objective ergometry test, which may introduce bias in the estimation of intervention effects.

### Conclusions

This study pioneered a social media–based intervention for patients with ACS. The findings demonstrated that the MCARE program was an effective approach to improving psychological distress, psychological stress, HRQoL, and several cardiovascular risk factors.

## Appendix

Multimedia Appendix 1 Structure and content of the mindfulness psycho-behavioral intervention (MCARE) program and supplemental results.

Multimedia Appendix 2 CONSORT eHEALTH checklist (V 1.6.1)

## Abbreviations

ACS: acute coronary syndrome
BP: blood pressure
GAD-7: 7-item Generalized Anxiety Disorder
GEE: generalized estimating equation
HRQoL: health-related quality of life
MCARE: mindfulness psycho-behavioral intervention
PHQ-9: 9-item Patient Health Questionnaire
RCT: randomized controlled trial

## Data Availability

The data sets generated during or analyzed during this study are available from the corresponding author on reasonable request.
